# Synaptic loss and progression in mice infected with *Angiostrongylus cantonensis* in the early stage

**DOI:** 10.1186/s12974-022-02436-8

**Published:** 2022-04-12

**Authors:** Kai-Yuan Jhan, Pi-Kai Chang, Chien-Ju Cheng, Shih-Ming Jung, Lian-Chen Wang

**Affiliations:** 1 Graduate Institute of Biomedical Sciences, College of Medicine, Chang Gung University, Taoyuan, 333 Taiwan; 2 Department of Parasitology, College of Medicine, Chang Gung University, Taoyuan, 333 Taiwan; 3 Department of Pathology, Chang-Gung Memorial Hospital, Chang-Gung Children Hospital at Linkou and Chang-Gung University, Taoyuan, 333 Taiwan; 4 Molecular Infectious Disease Research Center, Chang Gung Memorial Hospital, Taoyuan, Taiwan

**Keywords:** *Angiostrongylus cantonensis*, Cognitive function, Behavioral test, Pathological changes, Golgi staining, Neuronal death, Synaptic loss

## Abstract

**Background:**

*Angiostrongylus cantonensis* is also known as rat lungworm. Infection with this parasite is a zoonosis that can cause eosinophilic meningitis and/or eosinophilic meningoencephalitis in humans and may lead to fatal outcomes in severe cases. In this study, we explored the mechanisms of the impairments in the cognitive functions of mice infected with *A. cantonensis.*

**Methods:**

In infected mice with different infective intensities at different timepoint postinfection, loss and recovery of cognitive functions such as learning and memory abilities were determined. Neuronal death and damage to synaptic structures were analyzed by Western blotting and IHC in infected mice with different infection intensities at different timepoint postinfection.

**Results:**

The results of behavioral tests, pathological examinations, and Golgi staining showed that nerve damage caused by infection in mice occurred earlier than pathological changes of the brain. BDNF was expressed on 14 day post-infection. Cleaved caspase-3 increased significantly in the late stage of infection. However, IHC on NeuN indicated that no significant changes in the number of neurons were found between the infected and uninfected groups.

**Conclusions:**

The synaptic loss caused by the infection of *A. cantonensis* provides a possible explanation for the impairment of cognitive functions in mice. The loss of cognitive functions may occur before severe immunological and pathological changes in the infected host.

**Supplementary Information:**

The online version contains supplementary material available at 10.1186/s12974-022-02436-8.

## Introduction

*Angiostrongylus cantonensis* is the most important causative agent of eosinophilic meningoencephalitis in humans [1, 2]. The first documented human infection was recognized in Taiwan (Tainan Hospital) in 1944 after an autopsy of a 15-year-old boy who died of suspected eosinophilic meningoencephalitis. Approximately 10 live immature worms were discovered from the patient’s cerebrospinal fluid [3].

Although infected people with angiostrongyliasis are commonly recognized in Southeast Asia, the Caribbean, the Pacific Basin, and especially in China, the infection was recently found to be an emerging infectious disease and widespread throughout the world. By 2010, more than 2900 cases had been reported in approximately 30 countries [4–6]. Humans are a nonpermissive host of *A*. *cantonensis*. The infection is due to ingestion of raw or uncooked intermediate hosts (land snails, freshwater snails, or slugs) or paratenic hosts (crustaceans, frogs, fishes, or planarians) contaminated with infective third-stage larvae. People may also acquire infection by consuming vegetables polluted with infective larvae in the mucus trail of infected gastropods [7].

The brain is the important organ responsible for cognitive functions, including learning and memory formation. Brain lesions caused by internal interferences such as drugs, thrombosis, or invasions of pathogens may affect learning and memory [8, 9]. Parasitic infections have been observed to change the behavior of their hosts and facilitate their transmission and completion of life history. The fish *Rutilus lacustris* living in deep waters changes its habitat to shallow waters near the shore after infection with *Ligula intestinalis*. This behavior increases the chance of predation of the fish by waterfowl and enables the spread of the parasite to its avian final hosts [10]. Infection of bees with *Nosema ceranae* stimulates prematurity in development. The flight activity of premature bees is higher than that of normal bees, and the success rate of homing is decreased. However, these behavioral changes do not have a direct relation to the life history of the parasite [11].

In addition to behavioral changes, the memory of the host may also be affected by parasitic infections. Testing mice infected with three different intensities of *Toxocara canis* (*T. canis*) larvae in T-maze, water maze, and elevated plus maze assessments demonstrated poor spatial learning and memory in the high-dose group [12]. Using the water maze to determine spatial learning and memory ability, *Toxoplasma gondii* (*T. gondii*) infected mice were found to have significantly longer training days, swimming times, and swimming distances than their uninfected counterparts. These findings confirm that *T. gondii* infection affects spatial learning in mice [13].

Zhang et al. employed flow cytometry to analyze brain cells in BALB/c mice infected with *A. cantonensis*. Necrosis and apoptosis of astrocytes and microglia were found in brain tissue 21 days after infection, but did not cause neuronal death. However, the results of the water maze showed significant decreases in the spatial learning and memory abilities of the infected mice on day 14 postinfection [14]. Although *A*. *cantonensis* infection did not cause the death of neurons, it significantly affected the cognitive functions regulated by the neurons. Therefore, this study intended to explore the mechanisms of spatial learning and memory impairment in mice infected with *A. cantonensis.* Considering that BALB/c mice were susceptible to *A. cantonensis*, C57BL/6 mice with higher tolerance should be used to avoid affecting the accuracy of behavior-related tests due to mobility [15–17].

## Materials and methods

### Maintenance of the life cycle of *A*. *cantonensis*

By the modified Baermann filtration method, first-stage larvae were isolated from the feces of infected rats before infection with *B*. *glabrata* snails. On day 21, the infected snails were sacrificed, and third-stage larvae were recovered from snail tissues minced with a homogenizer (Cole-Parmer Instrument Co., USA). After digestion with artificial gastric juice (0.6% w/v pepsin, pH 2–3) at 37 °C for 45 min, the third-stage larvae were isolated and then inoculated into SD rats. On day 50 postinfection, first-stage larvae will be isolated from the feces of the infected rats.

### Experimental animals

For *A*. *cantonensis* life cycle maintenance, SD rats were purchased from the National Laboratory Animal Center (Taipei) or BioLASCO (Taipei). For experimental studies, C57BL/6 mice (7–8 weeks) were purchased from the National Laboratory Animal Center (Taipei) or BioLASCO (Taipei). These animals were cared for in the Animal Center of Chang Gung University. They were reared at a constant temperature and provided with food and drinking water ad libitum. All procedures involving animals and their care were reviewed and approved by the Chang Gung University Institutional Animal Care and Use Committee (IACUC Approval No.: CGU108-208).

### Experimental infection

After isolation from *B*. *glabrata* snails by the above method, the third-stage larvae were counted and removed by a glass capillary under a dissecting microscope. Each mouse was inoculated with 50 larvae by stomach intubation using a feeding tube.

### Y-maze test

This study was to investigate the changes in cognitive function of mice at specific timepoints after infection. The mice were divided into MWM group and NOR + Y-maze group. The Y-maze test was employed to test the effect of angiostrongyliasis on the spatial working memory performance of mice. There were 10 mice in each of the control group and the infection groups. A total of 40 mice were used in this experiment. The device consists of custom-made Y-mazes with three symmetrical and identical arms (15 × 5 × 40 cm). The tested mouse was placed at the end of a randomly assigned arm and allowed to freely navigate in the maze for 5 min. The sequence and number of arm entries were recorded using a video camera throughout the maze. The sequence triads in which all three arms are explored (including ABC, ACB, BAC, BCA, CAB, and CBA) are considered successful alternations with normal perception and short-term memory. The percentage of successful alterations is calculated as the number of successful alterations divided by the number of possible alternations (the total number of arm entries − 2). The total number of arm entries serves as an indicator of locomotor activity [18].

### Morris water maze test

The Morris water maze (MWM) test was used to assess the effect of angiostrongyliasis on spatial memory. There were 10 mice in each of the control group and the infection groups. A total of 40 mice were used in this experiment. The custom-made maze is a circular pool 120 cm in diameter and 40 cm in height filled with water at 25 ± 1 °C that is made opaque with the addition of milk. In the acquisition session, each mouse was given four trials per day and 4 days of training in total to find a hidden platform located 1.5 cm below the water surface. Each mouse was placed into the pool facing the wall, with a different starting point for each trial in which the direct route to the platform differed each time. The time required by the mouse to find and stand on the platform was recorded for up to 90 s. The mouse was allowed to stay on the platform for 30 s. It was then removed from the maze and placed in its cage. If the mouse did not find the platform within 90 s, the animal was placed on the platform for 30 s. The intertrial interval was at least 30 min. In the probe session, on day 5, the platform was removed from the pool. The mouse was tested in a probe trial for 60 s. Mouse swimming tracks were recorded using a TopScan automated tracking system (Clever Sys Inc., Virginia, USA) [18].

### Novel object recognition task

The novel object recognition (NOR) task was used to evaluate the effect of angiostrongyliasis on recognition memory. There were 10 mice in each of the control group and the infection groups. A total of 40 mice were used in this experiment. The NOR task consisted of three types of sessions: habituation, training, and testing. For habituation, the tested mouse was placed in a box for 10 min in the absence of test objects once per day for three consecutive days. On the fourth day, two identical objects were placed symmetrically on two opposite sides of the box. The mouse was allowed to explore the box for 10 min in the training session. Approximately 24 h after the training session, the mouse was allowed to explore the same box. However, one familiar object was replaced by a novel object. The animals were then allowed to explore freely for 10 min. The times spent exploring familiar or novel objects are recorded. Data were compiled up to 40 s of total exploration time. Object exploration was defined as a mouse touching or sniffing the object at a distance less than or equal to 2 cm. The discrimination index (DI) refers to the difference in exploration time for a novel and a familiar object divided by the amount of exploration of the novel and familiar objects [DI = (novel − familiar)/(novel + familiar)] [18].

### Collection of blood and brain specimens

From each mouse, blood samples were collected by cardiac puncture after complete anesthetization with isoflurane. The brain is then removed from the cranial cavity. Serum specimens were prepared from the blood samples by centrifugation and kept at − 80°. The brain is stored in 10% formalin solution.

### Preparation of sera

After cardiac puncture, blood samples were placed at room temperature for 2 h and then at 4 °C overnight. After centrifugation at 1300×*g* for 45 min, the supernatant was collected and kept at − 80 °C.

### Brain sections

After complete fixation in 10% formalin solution, each brain was cut into five sections: anterior cerebrum, lateral ventricles, third ventricle and hippocampus, posterior cerebrum, and fourth ventricle. These sections were then dehydrated with alcohol, embedded in paraffin, and cut into slices.

### Western blotting

There were 5 mice in each of the control group and the infection groups. Brain tissues were homogenized in ice-cold 1% SDS and protein concentrations were determined in triplicates using the Bradford protein assay. The extracts were mixed with Laemmli sample buffer and placed in a boiling water bath for 5 min. Proteins were separated by SDS–PAGE and blotted onto nitrocellulose membranes. Designated protein species (BDNF; cleaved caspase-3 for apoptosis; Beclin-1 for autophagy; RIP3 for necroptosis) (Abcam, ab108319, UK; Cell Signaling Technology, 9664, USA; Abcam, ab62557, UK; Abcam, ab56164, UK) were detected using their corresponding specific primary antibodies followed by horseradish peroxidase conjugated secondary antibodies. Protein immunoreactive signals are detected with the ChemiDocTM XRS + System (Bio-Rad, USA).

### Enzyme-linked immunosorbent assay

After coating wells of ELISA microplates with the somatic antigen preparation from the fifth-stage larvae at 4 °C overnight, the wells were then washed with a blocking buffer before adding serum specimens (containing the primary antibodies) from C57BL/6 mice sacrificed at different timepoints. There were 5 mice in each of the control group and the infection groups. The excess amounts were removed by washing and secondary antibodies (goat anti-mouse IgA/G/M; goat anti-mouse IgG; goat anti-mouse IgM) (KPL, 07511807, USA; Sigma-Aldrich, A3562, USA; KPL, 07511803, USA) conjugated with alkaline phosphatase were then added. Color was then developed after adding a substrate and reactions were terminated by adding a stop solution. Readings were measured at OD 405 nm.

### Immunochemical staining

Deeply anesthetized mice were transcardially perfused with PBS followed by 4% paraformaldehyde in PBS. Fixed brains were sliced into 30 µm sections. There were 5 mice in each of the control group and the infection groups. PSD95, SYN, NeuN (Cell Signaling Technology, 3409, USA; Abcam, ab32127, UK; Abcam, ab128886, UK) were stained in each of the five brain sections from each animal. Image J was used to threshold the stained brain tissue and to analyze the color ratio of the fixed area in the imaging.

### Hematoxylin and eosin staining

Each brain section was sliced into 3 µm sections and treated with xylene to remove paraffin and then stained with Mayer’s hematoxylin solution and eosin solution. The slice was mounted on a microscopic slide using mounting medium.

### Histopathological examination

Regions of the mouse brain were divided into anterior cerebrum, lateral ventricles, third ventricle and hippocampus, posterior cerebrum and fourth ventricle, and cerebellum. Eight pathological changes were employed to determine the degree of brain damage post-infection. These changes included eosinophilic meningitis, brain necrosis, choriomeningitis, size/finding of larvae, meninges congestion, perivascular cuffing, encephalitis, and hemorrhage. After determining the area of each change in the slices, medians were calculated and employed as a cutoff point for severity. Negative results were scored 0, scores of 1 represented those below or equal to the median, and scores of 2 indicated those above the median. A score of severity for each pathological change was calculated by each group of mice [19].

### Golgi staining

After removal from the cranial cavities of the mice, the brains were quickly removed and prepared for Golgi impregnation using the FD Rapid Golgi Stain Kit (FD Neurotechnologies, Baltimore, MD). There were 5 mice in each of the control group and the infection groups. After incubating in Golgi-Cox for 17 days at room temperature in the dark, the brains received solution C at 4 °C for 3 days before embedding in OCT and storage at − 80 °C. sections (150 µm) were transferred into the staining solution for 10 min before dehydration, cleaning in xylene, and coverslipping with DPX mounting medium (VWR International, Leuven, Belgium). Images were captured on an Olympus BX53 microscope (100X magnification). Spines from granular neurons were counted and normalized against the total dendritic length.

### TUNEL assay

For the neuronal apoptosis assay, frozen brain sections were stained using the terminal deoxynucleotidyl transferase dUTP nick end labeling (TUNEL) kit (AP 11684809910; Roche Life Science, Switzerland) to detect DNA fragmentation. There were 5 mice in each of the control group and the infection groups. We used image J to threshold the stained brain tissue and to analyze the color ratio of the fixed area in the imaging.

### Statistical analyses

Data are expressed as the mean ± standard deviation. Differences among the groups were analyzed by one-way ANOVA, one-way repeated measures ANOVA, two-way ANOVA, and two-way repeated measures ANOVA.

## Results

### Behavioral test of C57BL/6 mice infected with *A. cantonensis*

**Fig. 1:**
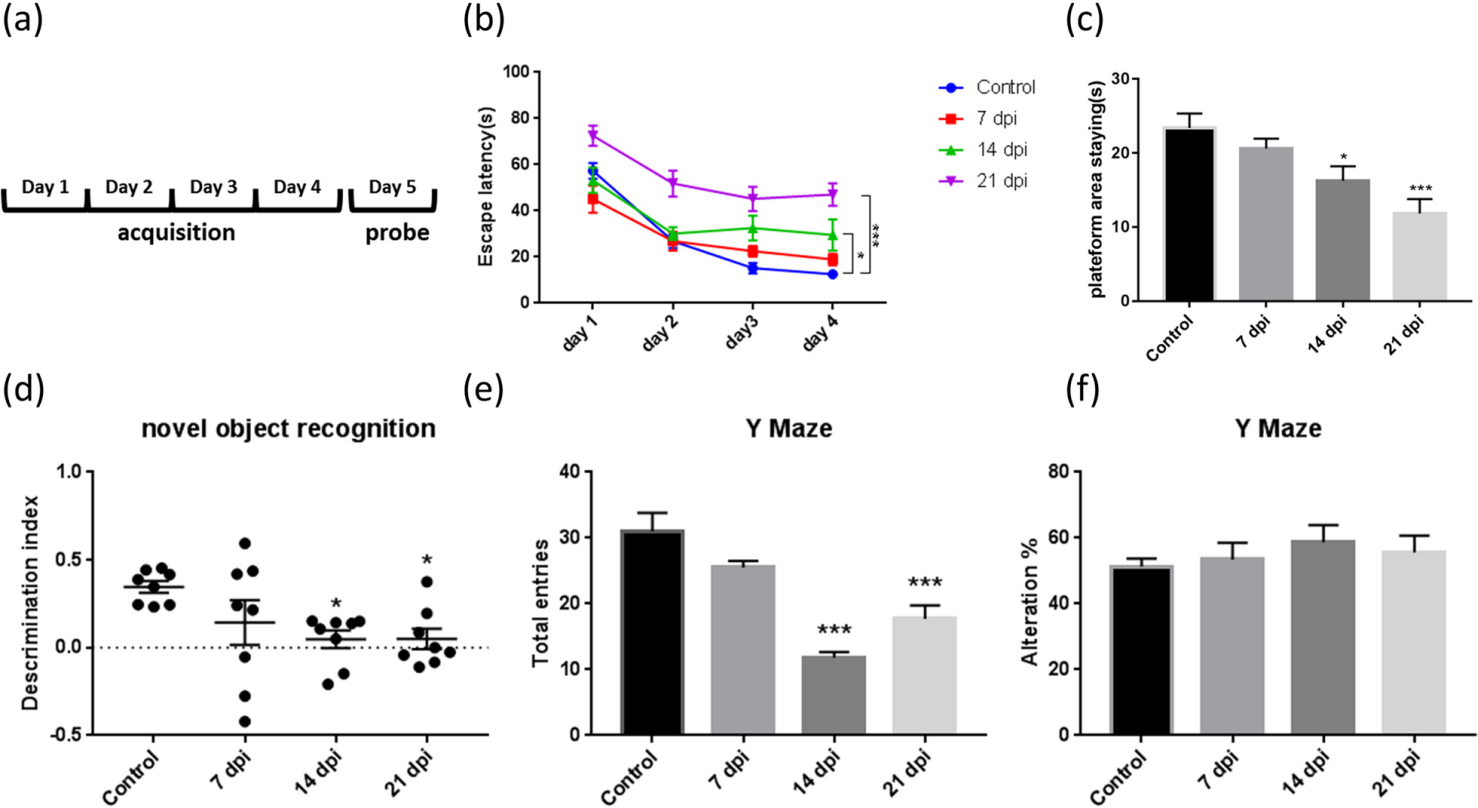
Evaluation of brain nerve function of C57BL/6 mice before and after *Angiostrongylus cantonensis* infection by behavioral tests. Different behavioral tests were used to evaluate the neurological changes of C57BL/6 mice at different timepoints after infection. **a** Morris water maze was divided into acquisition test (spatial learning) on the first 4 days and the probe test (spatial memory) on the 5th day. **b** In the acquisition test phase of Morris water maze, the time for mice to reach the platform decreased with the increase of the number of learning days. After 14 days and 21 days of infection, the spatial learning ability of C57BL/6 mice was significantly reduced (*P < 0.05, ***P < 0.001). **c** In the probe test stage of Morris water maze, the time that C57BL/6 mice stayed in the target quadrant decreased with the increase in the number of days of infection, indicating that the spatial memory ability of the mice was affected by the infection, and significantly decreased after 14 days and 21 days of infection (*P < 0.05, ***P < 0.001). **d** NOR task was used to detect new things recognition memory in mice. C57BL/6 mice in the 14-day and 21-day infection groups cannot distinguish new and old things, and their ability to recognize new things and memory was significantly reduced (*P < 0.05, *P < 0.05). Y maze was used to evaluate the spatial working memory of mice. **e** Total number of times the mice entered each arm was recorded. The total number of entries decreased significantly after 14 days and 21 days after infection, indicating that the mice's desire to explore after infection was reduced (***P < 0.001, ***P < 0.001). **f** There was no significant difference in working memory ability between different groups of mice before/after infection. The data were analyzed by One-Way ANOVA and Two-Way ANOVA

The Morris water maze was divided into an acquisition test (spatial learning) on the first 4 days and a probe test (spatial memory) on the 5th day (Fig. 1a). In the acquisition test, the time for mice to reach the platform decreased with the increase in the number of learning days. The spatial learning ability of C57BL/6 mice was significantly reduced on day 14 postinfection (Fig. 1b). In the probe test, the time that C57BL/6 mice stayed in the target quadrant decreased with the increase in the number of days of infection, indicating that the spatial memory ability of the mice was affected by the infection, and it decreased significantly on day 14 postinfection (Fig. 1c).

NOR task was used to evaluate the ability of mice to recognize new things before and after infection with *A. cantonensis*. The results showed that the ability of the mice to recognize new things was significantly reduced on day 14 postinfection (Fig. 1d). The Y maze was used to explore the effect of *A. cantonensis* infection on the spatial working memory of C57BL/6 mice. The number of infected mice entering the arms was significantly reduced on day 14 postinfection (Fig. 1e). There was no significant change in the working memory ability of infected mice (Fig. 1f). The open field test results showed that *A. cantonensis* infection did not affect the mobility of the mice (Additional file 1: Fig. S1).

In summary, cognitive function related to the long-term memory of mice was impaired in the early stages of infection. In the Y maze, it was found that *A. cantonensis* infection may have an effect on the desire to explore, but it has no effect on short-term memory ability.

### Changes in nerve structure in C57BL/6 mice infected with *A. cantonensis*

**Fig. 2:**
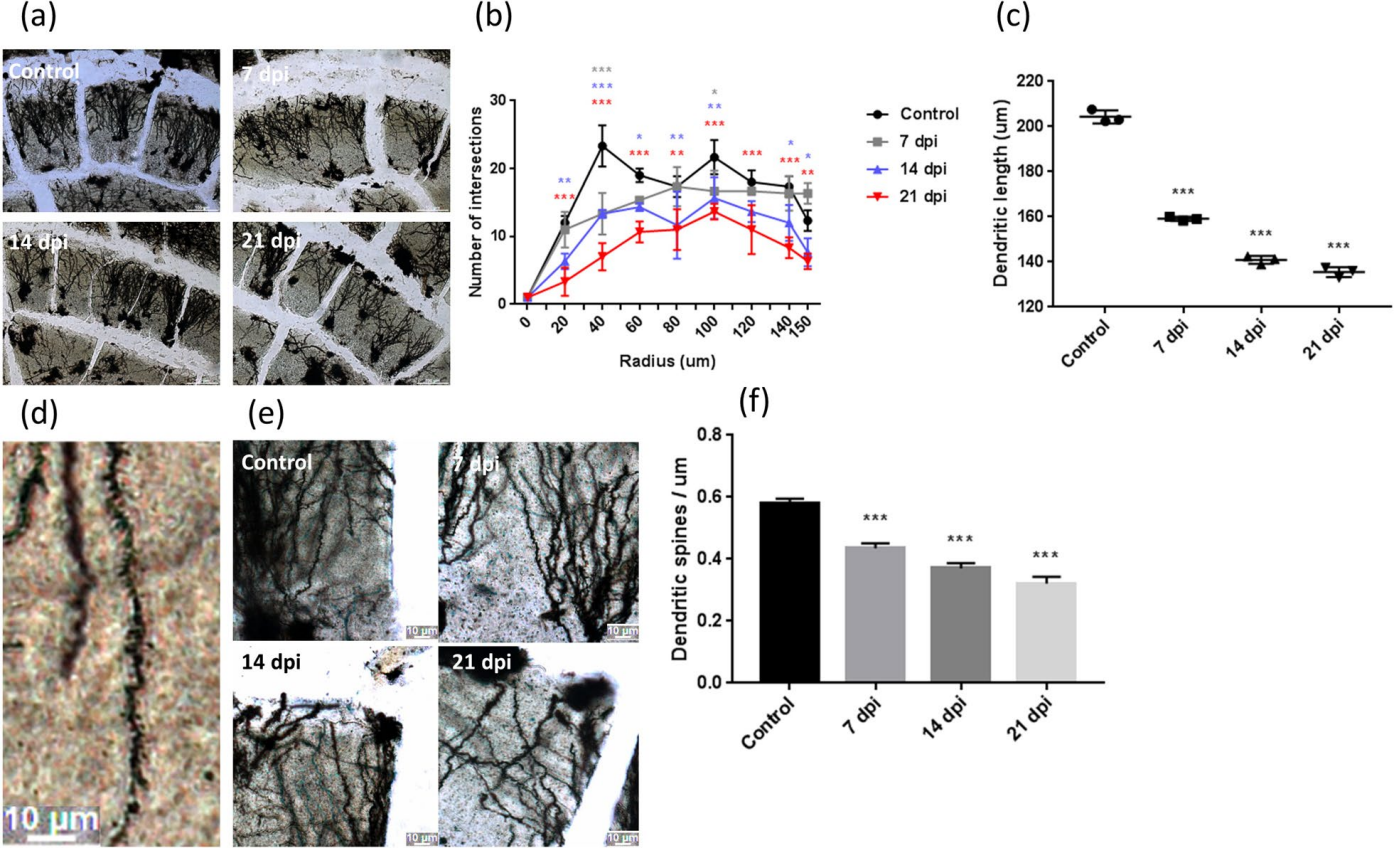
Induction of dendrite damages in CA1 pyramidal neurons in *A. cantonensis* infected mice detected by Golgi staining. **a** Nerve distribution in the hippocampus of C57BL/6 mice tends to decreased with the increased of days of infection (bar = 100 μm). **b** Total number of dendrite-ring intersections decreased with time (*P < 0.05, **P < 0.01, ***P < 0.001). **c** Length of dendrites decreased significantly on day 7 postinfection (***P < 0.001). **d** Dendritic segments showing spine morphology in infected mice (bar = 10 μm). **e**, **f** Dendritic spine density significant decreased on day 7 postinfection (***P < 0.001) (bar = 10 μm). The data were analyzed by One-Way ANOVA

Dendrite damage in the hippocampal neurons of infected mice was detected by Golgi staining. The nerve distribution in the hippocampus of C57BL/6 mice tended to decrease with increasing days of infection (Fig. 2a). The total number of dendritic ring intersections decreased as the infection time increased. The distribution of dendritic intersections was observed; the reduced part was mainly located on the side close to the neuron, and the distal end only decreased significantly in the late stage of infection. Dendritic intersections represent the complexity of nerve structure and the dynamic range of nerves (Fig. 2b). The length of dendrites decreased significantly on day 7 postinfection (Fig. 2c). Dendritic segments showed the spine morphology in infected mice (Fig. 2d). The dendritic spine density significantly decreased on day 7 postinfection (Fig. 2e, f). The nerve dendrite structure of infected mice was damaged in the early stages of infection.

### Effect of *A. cantonensis* infection on the expression of synaptic proteins in C57BL/6 mouse brains

**Fig. 3:**
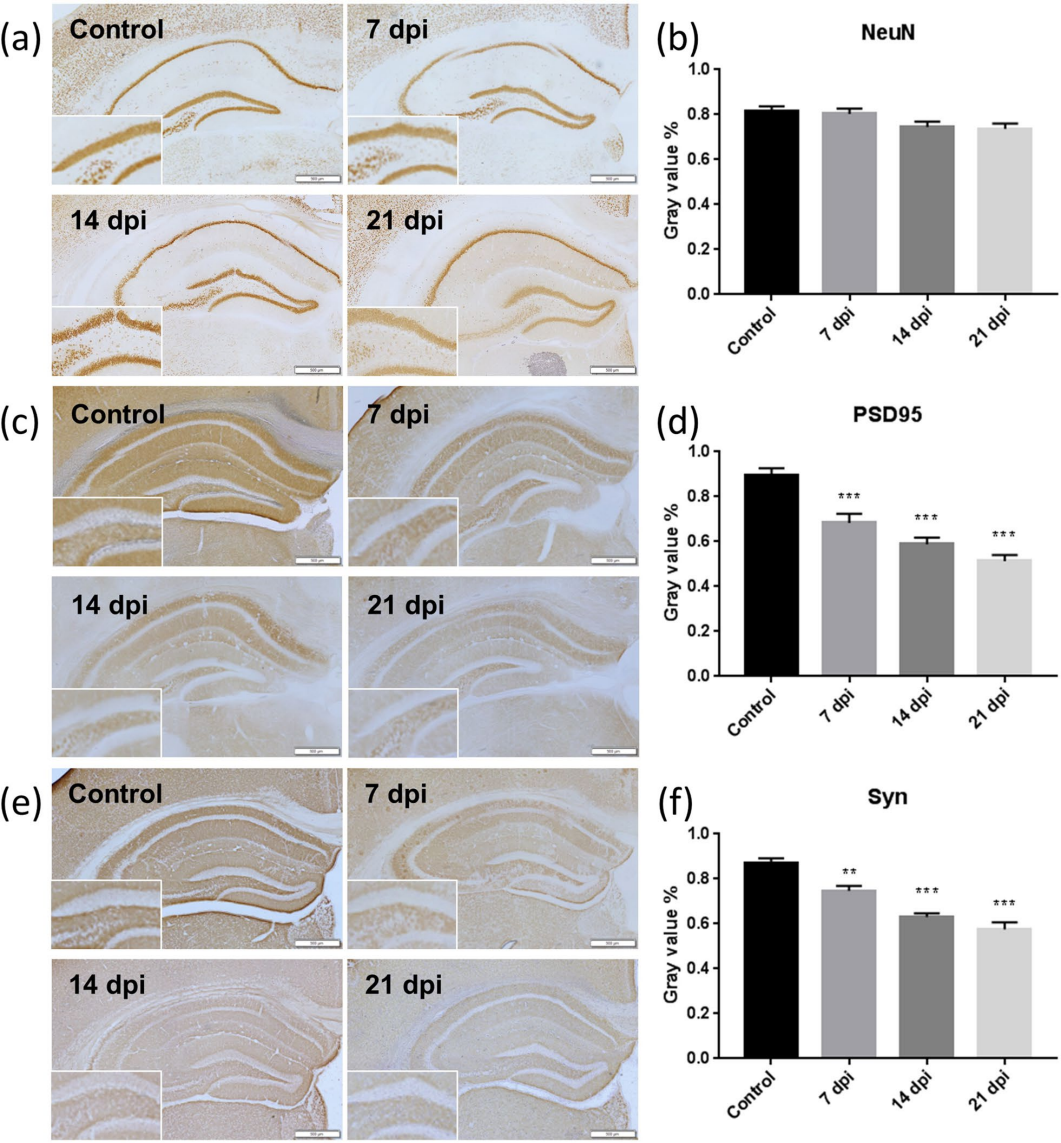
Changes in the level of NeuN and synaptic proteins expression in hippocampus of mice infected with *A. cantonensis* detected by immunohistochemisry. The brains of C57BL/6 mice infected with *A. cantonensis* were analyzed by immunohistostaining. **a**, **b** There was no significant difference in NeuN expression in the hippocampus of mice before and after infection (bar = 500 μm). **c**, **d** Level of PSD95 significantly decreased on day 7 postinfection (***P < 0.001) (bar = 500 μm). **e**, **f** Level of synaptophysin significantly decreased on day 7 postinfection (**P < 0.01) (bar = 500 μm). The data were analyzed by One-Way ANOVA

**Fig. 4:**
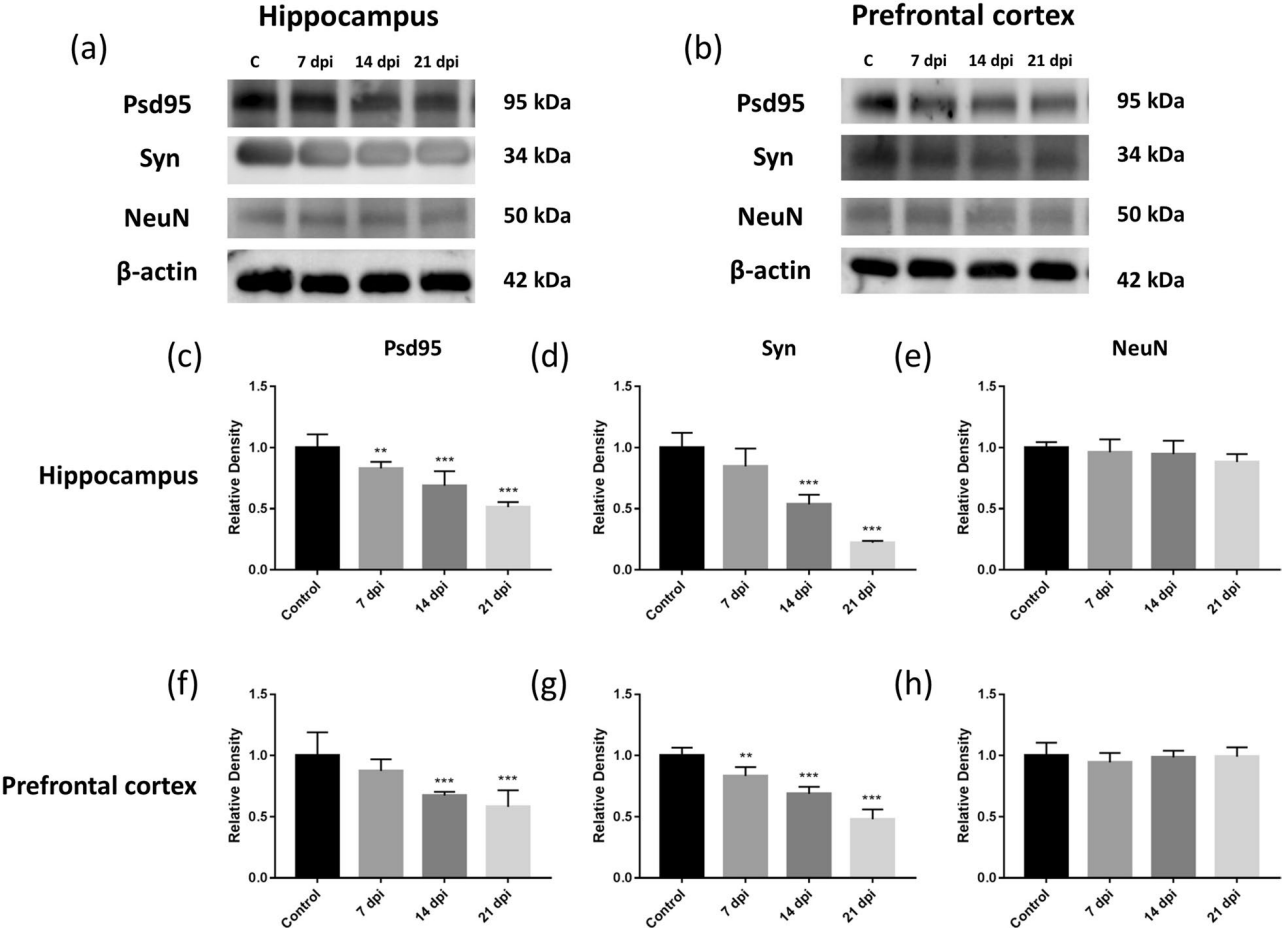
Changes in the level of PSD95, synaptophysin and NeuN expression in hippocampus and prefrontal cortex of mice infected with *A. cantonensis* detected by Western blotting. **a**, **b** Level of PSD95, synaptophysin and NeuN expression in hippocampus and prefrontal cortex of mice infected with *A. cantonensis* were detected by Western blotting. **c**, **d** Level of PSD95 and synaptophysin in the hippocampus of infected mice significantly decreased on day 7 postinfection (**P < 0.01). **e** There was no significant difference in NeuN expression in the hippocampus of mice before and after infection. **f**–**h** Expression of synaptic protein in prefrontal cortex of infected mice has the same trend as the hippocampus. The data were analyzed by One-Way ANOVA

Changes in the expression levels of PSD95, synaptophysin, and NeuN in the hippocampus of mice infected with *A. cantonensis* over time were determined by immunohistochemistry. The levels of PSD95 and synaptophysin significantly decreased on day 7 postinfection (Fig. 3d, f). NeuN is specific to the neuronal cell nucleus, so it is used to label neuronal cells. There was no significant decrease in NeuN expression at different timepoints after infection (Fig. 3b). These findings indicated that *A. cantonensis* infection may cause damage to nerve synapses but does not cause the death of neurons. The levels of PSD95, synaptophysin and NeuN expression in the hippocampus of mice infected with *A. cantonensis* were detected by Western blotting (Fig. 4a). The expression levels of PSD95 and synaptophysin decreased significantly after infection (Fig. 4c, d), while NeuN expression was not affected by *A. cantonenesis* infection (Fig. 4e). Then, we evaluated the expression levels of PSD95, synaptophysin and NeuN in the prefrontal cortex (Fig. 4b). The prefrontal cortex is also the functional area of the brain that regulates cognition. The expression levels of PSD95 and synaptophysin decreased significantly after infection (Fig. 4f, g), while NeuN expression was not affected by the infection (Fig. 4h) (Additional file 2: Fig. S2).

### Brain pathological changes and survival rates of C57BL/6 mice infected with *A. cantonensis*

**Fig. 5:**
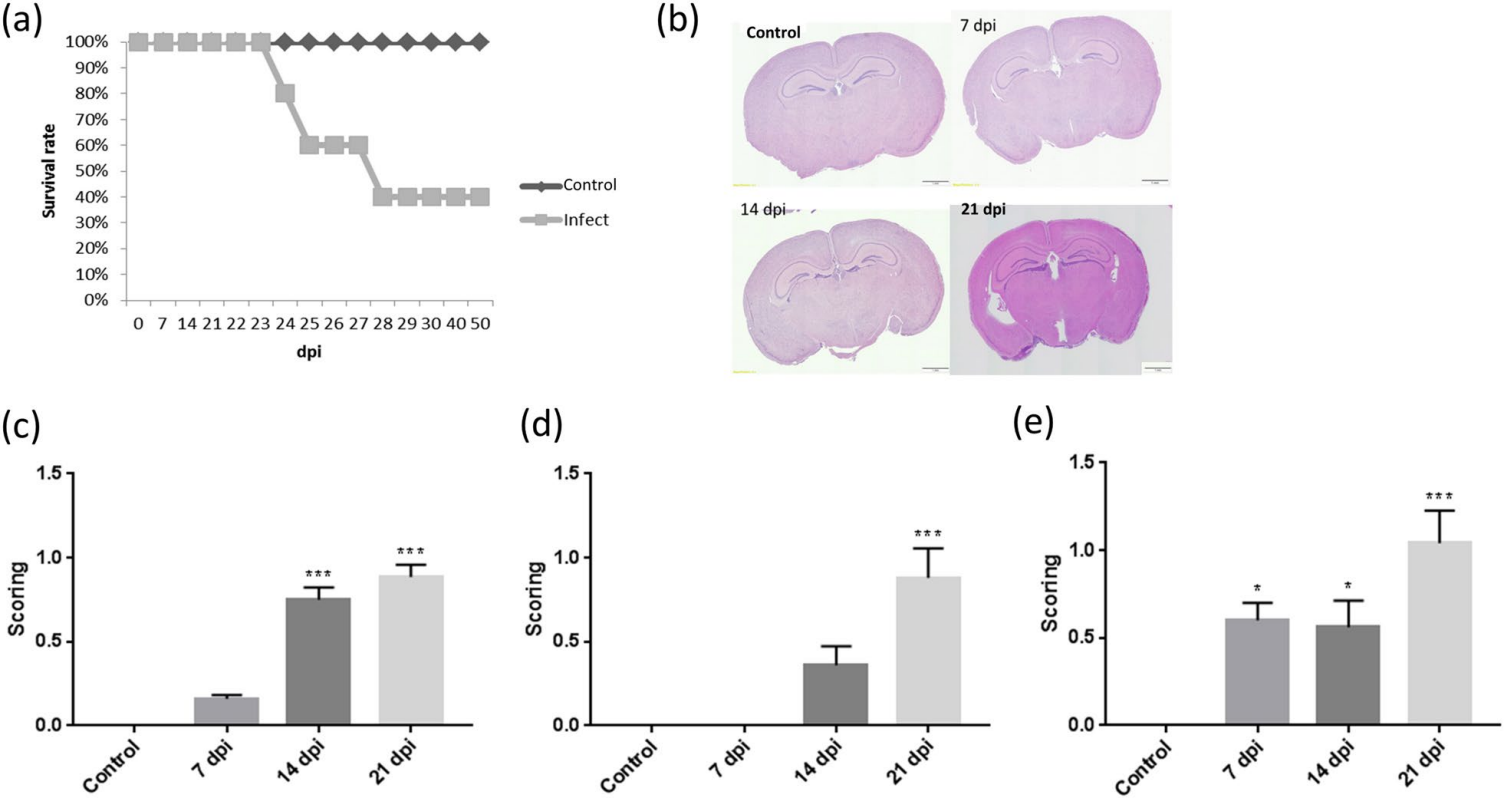
Pathological changes in brain section and survival rates of infected mice. **a** Infected mice started to die on day 24 postinfection. **b** C57BL/6 mice have different brain pathological phenomena at different timepoints after infection with *A. cantonensis*, and they have more serious symptoms on day 21 postinfection (bar = 1 mm). **c** Comparison of pathological findings in the cerebral parenchyma of infected mice at different timepoints, including eosinophilic meningitis, encephalitis, congestion, hemorrhage, perivascular cuffing, larvae finding, choroid plexus inflammation, tissue necrosis. The scoring has increased significantly on days 14 and 21 postinfection (***P < 0.001, ***P < 0.001). **d** Comparison of encephalitis in the cerebral parenchyma of infected mice at different timepoints. The scoring has increased significantly on day 21 postinfection (***P < 0.001). **e** Scoring of presence of larvae in the cerebral parenchyma of infected mice. The data were analyzed by One-Way ANOVA

Pathological changes (eosinophilic meningitis, hemorrhage, larvae, encephalitis) in the brain and survival rates of infected mice were compared at different timepoints (Fig. 5). Infected mice died starting on day 23 postinfection (Fig. 5a). The pathological findings of infected mice had higher scores on day 14 postinfection (Fig. 5b, c). After day 21, the severity of encephalitis increased in the infected mice (Fig. 5d). Scoring of the presence of larvae in the cerebral parenchyma of infected mice was higher on day 21 postinfection (Fig. 5e). The results demonstrated that pathological changes mainly appeared in the late stage of *A. cantonenesis* infection, and there was no serious inflammatory reaction in the early stage of infection.

**Fig. 6:**
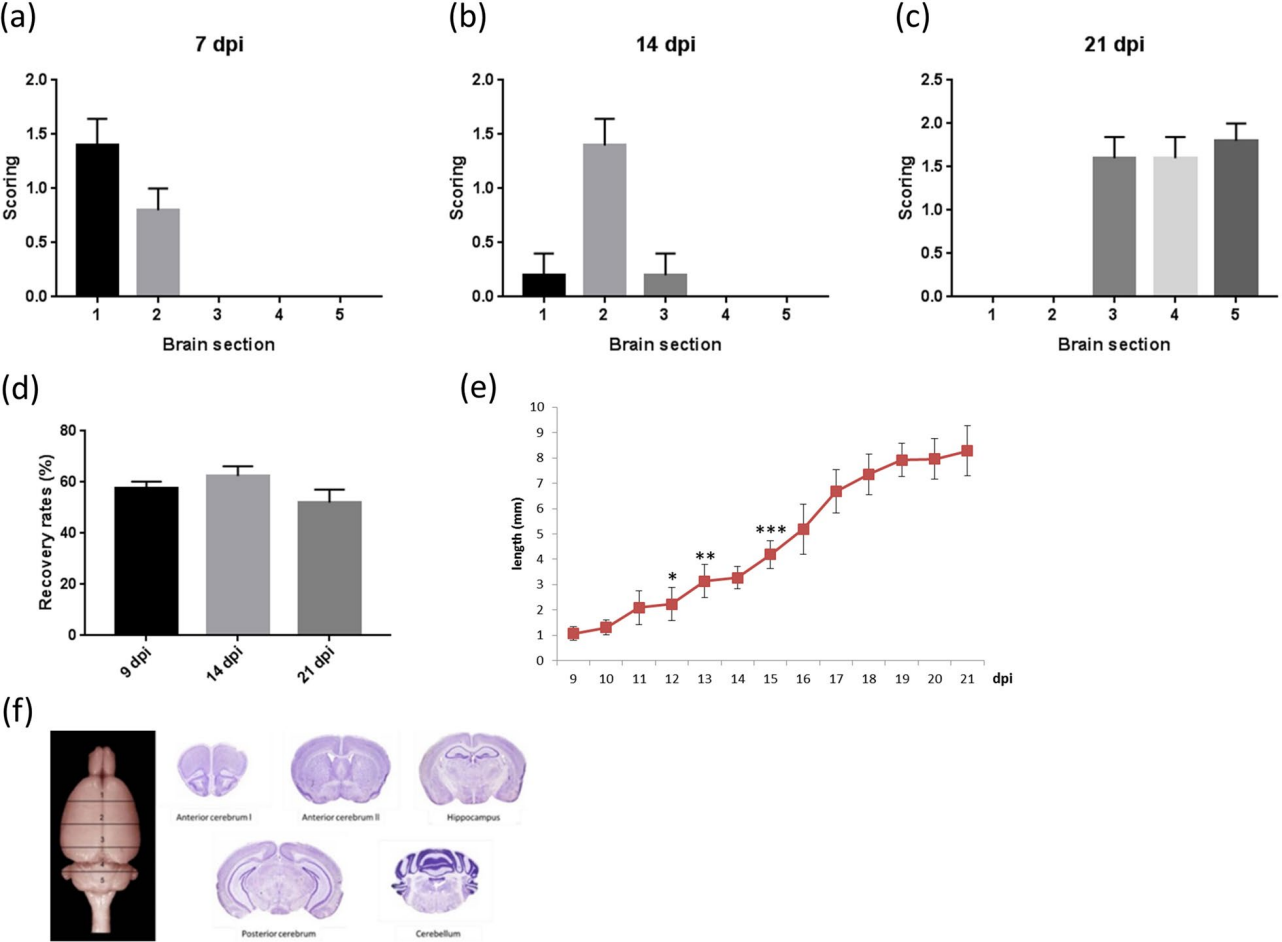
Presence of larvae in different section of brain. **a**–**c** Location of larvae in the brain at different timepoints after infection. **d** There were no differences in the recovery rates of larvae from the mice brain at different timepoints after infection. **e** Measure the length of the larvae, the length increased significantly on day 12 postinfection (*P < 0.05, **P < 0.01, ***P < 0.001). **f** Sections of brain tissues. The data were analyzed by One-Way ANOVA

We wanted to know whether the migration of the larvae in the brain caused mechanical damage to the nerve structure. The location of larvae in the brain at different timepoints after infection was estimated (Fig. 6). The larvae were mainly distributed in the first area of the brain section containing the olfactory bulb and the prefrontal cortex on day 7 postinfection (Fig. 6a), and the larvae were mainly distributed in the second area of the brain section containing the striatum on day 14 postinfection (Fig. 6b). On day 21 postinfection, the larvae migrated to the back of the brain and were active in the third, fourth, and fifth areas of the brain section, including the hippocampus and cerebellum (Fig. 6c). There was no difference in the recovery rates of larvae at different timepoints after infection (Fig. 6d). The larvae grew in length as the number of days of infection increased, and the length increased significantly on day 12 postinfection (Fig. 6e). The results showed that *A. cantonensis* infection caused the impairment of cognitive function and the destruction of synaptic structure in the early stage of infection, but the larvae had not yet migrated to the hippocampus and caused mechanical damage.

### Expression of cell death-related proteins in the brains of C57BL/6 mice infected with *A. cantonensis*

**Fig. 7:**
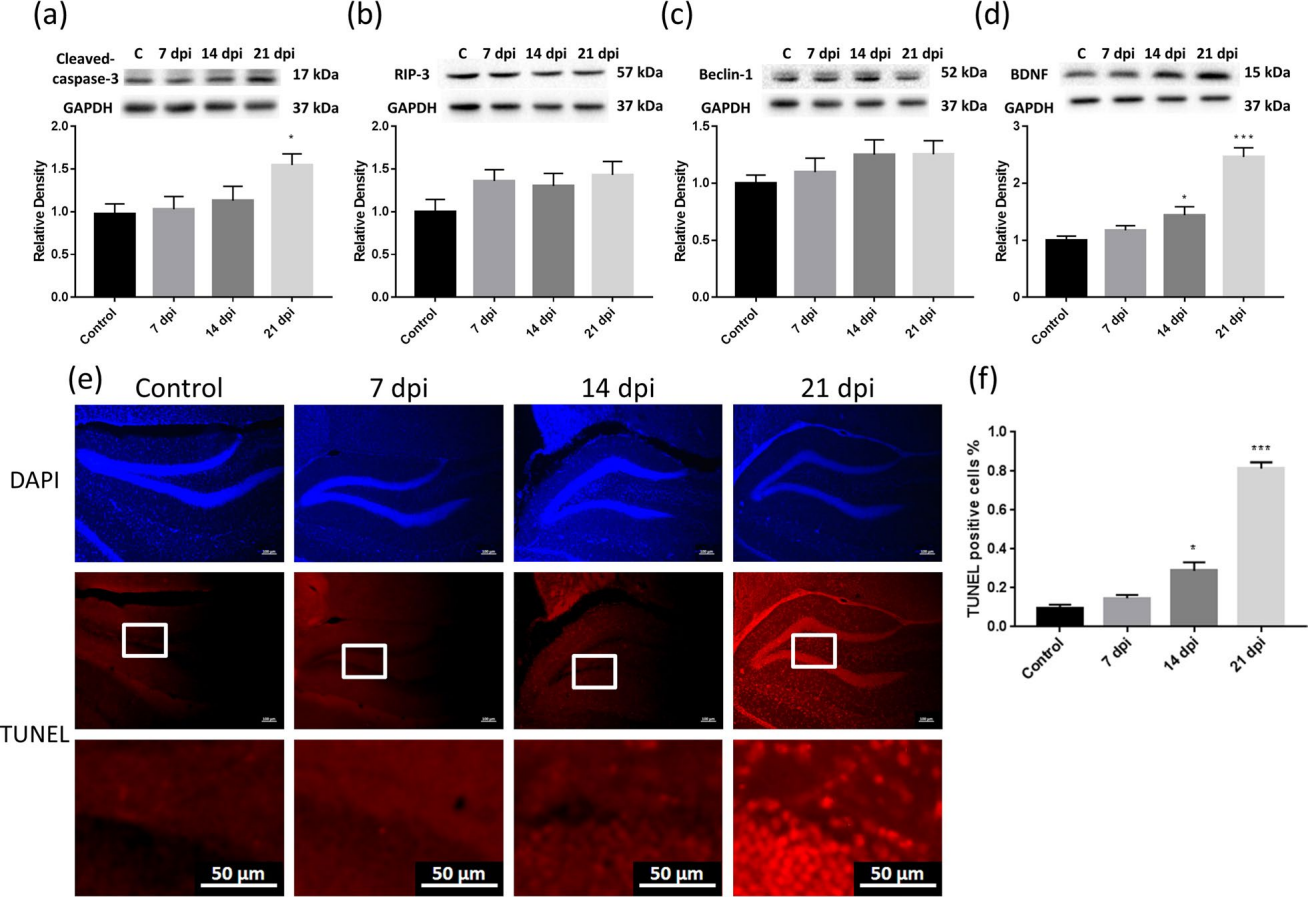
Expression of cell death-related proteins in the brain of C57BL/6 mice infected with *A. cantonensis*. Western blotting was used to detect the expression of brain cell death-related proteins. **a** Expression of BDNF in the brain of infected mice showed significant manifestations on day 14 postinfection (*P < 0.05). **b** Apoptotic protein cleaved-caspase3 in the brain of infected mice increased significantly on day 21 postinfection (*P < 0.05). **c**, **d** There was no significant difference on the level of necroptotic protein RIP-3 and autophagy-related protein Beclin-1 before and after infection in mice. **e** Examples of the distribution and amounts of TUNEL positive cells by microscopic viewing (bar = 100 μm, 50 μm). **f** Mean fraction of TUNEL staining-positive cells to the number of available cells (*P < 0.05; ***P < 0.001). The data were analyzed by One-Way ANOVA

The expression levels of BDNF, cleaved caspase-3, RIP3, and Beclin-1 in the hippocampus of mice infected with *A. cantonensis* over time were determined by Western blotting. The expression of BDNF significantly increased on day 14 postinfection (Fig. 7a), and the expression of cleaved caspase-3 significantly increased on day 21 (Fig. 7b).

The TUNEL assay was used to confirm cell death in the brains of mice before and after infection. Cell death increased significantly on day 21 postinfection (Fig. 7e, f). The results showed that the nerve structure damage and cognitive dysfunction caused by *A. cantonensis* infection in the early stage were not caused by cell death.

### Peripheral immune response of C57BL/6 mice infected with *A. cantonensis*

**Fig. 8:**
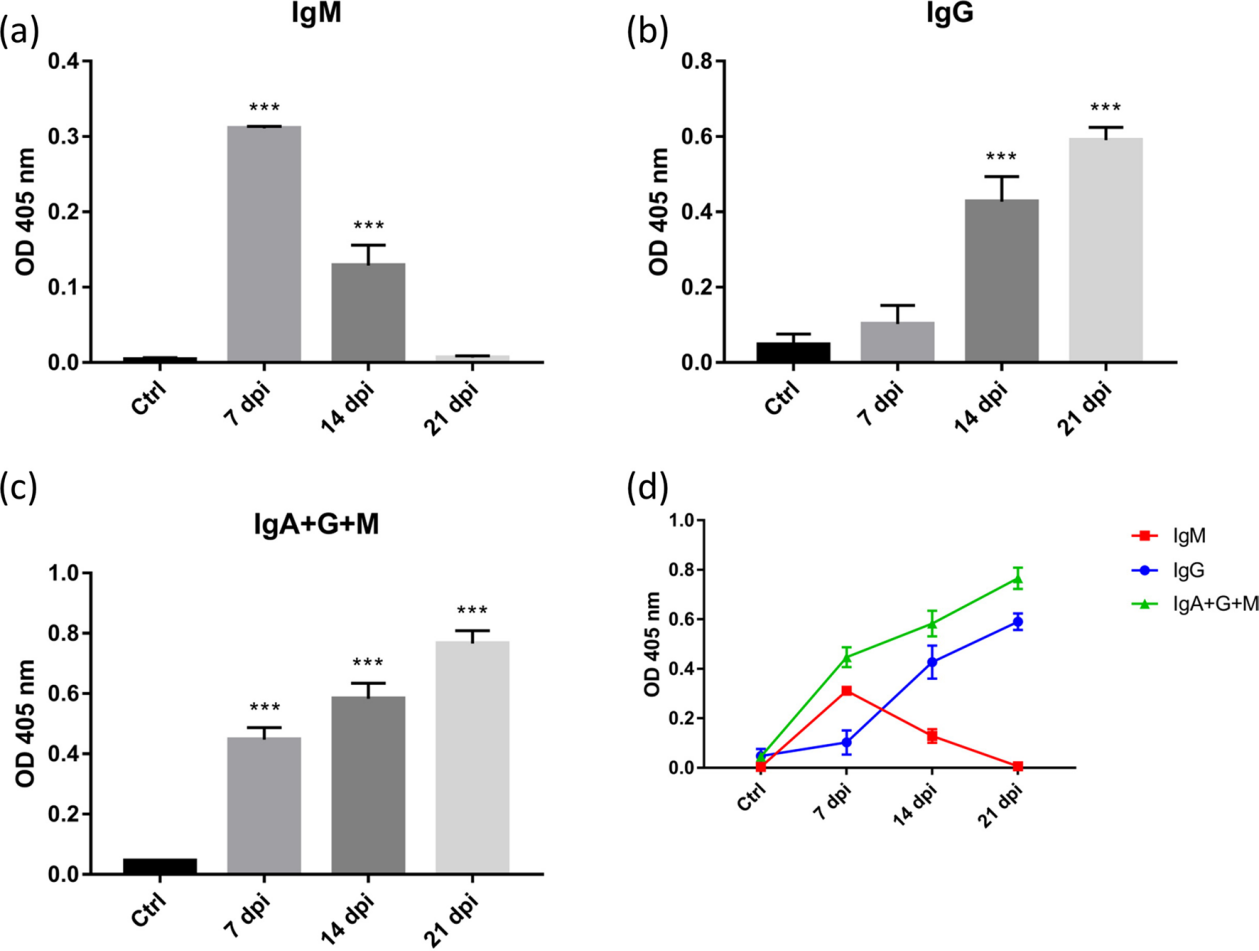
Peripheral immunoglobulin expressions in C57BL/6 mice infected with *A. cantonensis*. The expression of immunoglobulin in the serum of C57BL/6 mice infected with *A. cantonensis* was detected by ELISA. **a** IgM increased significantly after 7 days of infection, but gradually slowed down after 14 days of infection (***P < 0.001). **b** IgG increased significantly after 14 days of infection (***P < 0.001). **c** IgA + G + M was significantly manifested 7 days after infection, and it increased with the increase of days of infection (***P < 0.001). **d** At the initial stage of infection with *A. cantonensis*, IgM in the serum of mice was significantly manifested. As the infection time increases, IgM will decrease. After 14 days of infection, IgG will be abundantly expressed, and it will increase with the increase in the number of days of infection. The data were analyzed by One-Way ANOVA

The expression of peripheral immunoglobulin in the serum of C57BL/6 mice infected with *A. cantonensis* was detected by ELISA. IgM increased significantly on day 7 postinfection but gradually slowed down on day 14 postinfection (Fig. 8a). IgG increased significantly on day 14 postinfection (Fig. 8b). IgA + G + M levels had increased significantly on day 7 postinfection and continued to increase as the number of days of infection increased (Fig. 8c). In the early stage of *A. cantonensis* infection, the peripheral immunity of the mouse is expressed by IgM. As the infection time increased, it was highly expressed by IgG in the late stage (Fig. 8d).

## Discussion

The water maze was used to evaluate the spatial learning and memory abilities of C57BL/6 mice infected with *A. cantonensis*. The results showed that the spatial learning and memory abilities of the mice were impaired on day 14 postinfection. The NOR task and Y maze were used to test the spatial memory and working memory abilities of the infected mice. The NOR task results showed that the memory ability of the mice decreased significantly on day 14 postinfection. However, the Y maze test showed that there were no significant differences between the mice before and after infection. These findings indicate that *A. cantonensis* infection may not have much impact on working memory. *A. cantonensis* larvae invade the host's brain and mainly migrate between blood vessels and ventricles. Around the hippocampus is the third ventricle, and the prefrontal cortex is the brain parenchyma area. Therefore, it can be explained that the damage to the prefrontal cortex of the brain is lower than that of the hippocampus, and the impact on working memory is also relatively slight.

The survival rate and brain pathology of C57BL/6 mice were also analyzed in this study. The mice began to die on day 23 postinfection. Eight pathological changes were analyzed, including meningitis, congestion, hemorrhage, larval findings, choroid plexitis, perivascular cuffing, encephalitis, and brain necrosis. The overall pathological changes increased on day 14 postinfection. Analysis of encephalitis showed a significant increase on day 21 postinfection. Previous studies have also found that the larvae moved from the forebrain to the cerebellum with the time of infection. The larvae mostly appeared in the cavity rather than the brain parenchyma.

The density of dendritic spines was positively correlated with the formation of memory. An increased density of dendritic spines may strengthen the formation of memory. Aging or Alzheimer’s disease may cause a reduction in memory by decreasing the density of dendritic spines [20]. Moreover, spatial memory and working memory have also been related to the density of dendritic spines [21]. Golgi staining analysis of the hippocampus of the mice after behavioral tests showed that the density of dendritic spines on the nerve synapse decreased significantly on day 7 postinfection. The number of dendrite-ring intersections also decreased with the time of infection. *A. cantonensis* caused synaptic loss in the early stages of infection. The results of this study showed that the dendritic structure of the hippocampus began to be damaged in the anterior and middle segment of the mouse in the initial stage of infection, but the distribution of the posterior dendrites has not been affected. As the infection time increased, the overall structure of the dendrites was severely damaged.

Rats subjected to water maze training induced the rapid recruitment of NMDAR subunits and PSD95 to synaptic lipid rafts, which were associated with the formation of memory [22]. Environmental enrichment could be a potent cognitive enhancer for aged females, the mechanism of which was to enhance spatial memory and increase synaptophysin levels in the hippocampus and frontoparietal cortex [23]. IHC staining was used to analyze synaptic proteins in the mouse brain and the neuron marker NeuN. The synaptic proteins PSD95 and synaptophysin showed significant decreases on day 7 postinfection, while NeuN showed no significant difference before and after infection in mice. These findings suggest that *A. cantonensis* infection may cause damage to nerve synapses but does not cause the death of neurons. Moreover, *A. cantonensis* caused synaptic loss in the early stage of infection, which in turn reduced cognitive functions.

Synaptic loss is currently considered to be the most relevant factor for cognitive decline in neurodegenerative diseases, such as Alzheimer's disease (AD). Antibodies against the synaptic terminals—synapsin-1 and synaptophysin—are used as synaptic markers in the hippocampal complexes of AD patients and non-dementia control subjects. Compared with the control brain, all AD patients had significantly less synaptic staining in the outer half of the molecular layer of the dentate gyrus. In addition, it has also been found that synaptic loss is an early event in the disease process. Intrinsic factors (such as elevated calcium ions) and external factors (such as amyloid or Tau pathology-induced microglia phenotype transformation) can destroy the stability of dendritic spines and may particularly cause synaptic loss [24–26]. Studies have pointed out that neuroinflammation and excitotoxic damage will also reduce synaptic markers in the rat brain [27].

Previous studies have found that the excretory/secretory products (ESPs) of fifth-stage larvae of *A. cantonensis* can prompt mouse brain astrocytes to activate the Sonic hedgehog (Shh) pathway, reduce oxidative stress, induce autophagy, and inhibit cell apoptosis [28, 29]. Through the activated Shh pathway, the expression of nuclear factor-κB (NF-κB) was increased. The function of NF-κB is to regulate inflammation and promote the secretion of proinflammatory genes, including cytokines and chemokines, such as IL-1β and TNF-α. The ESP of fifth-stage larvae of *A. cantonensis* stimulated the activation of mouse brain astrocytes and the production of inflammation-related cytokines through the Shh pathway and NF-κB [30–32].

Infection with *A. cantonensis* caused damage to the nerve structure in the early stage of infection and affected cognitive function. Early infected larvae did not appear around the hippocampus. The symptoms of brain inflammation did not increase significantly until 21 days after infection. The results indicate that the larvae have related factors (e.g., excretory/secretory product, ESP) that may cause the loss of synapses and cognitive function at the initial stage of infection, rather than mechanical damage and inflammatory pathology.

Gosnell WL et al. suggested the role of eosinophils in angiostrongyliasis. Eosinophils have also been shown to store, produce, and release neurotrophins, such as BDNF. It has been suggested that locally produced BDNF in the CNS mitigates inflammation-dependent neuronal damage [33]. Studies have pointed out that traumatic brain injury (TBI) can activate the BDNF/TrkB pathway to cause changes in expression of BDNF mRNA [34]. The results of clinical study showed that in the CSF of TBI patients, there was a higher expression of BDNF [35]. The infection of *A. cantonensis* cause eosinophilic meningoencephalitis, and a large amount of eosinophil infiltration may also increase the expression of BDNF. Western blotting was used to analyze the expression levels of the mouse brain neurotrophic factor BDNF, the cell death-related apoptosis factor cleaved caspase-3, the necroptosis factor RIP3, and the autophagy factor Beclin-1. The results showed that the brains of mice were damaged on day 14 postinfection and that BDNF increased significantly. Regarding cell death-related factors, only the apoptotic factor cleaved caspase-3 increased significantly on day 21 postinfection, while the necroptosis factor RIP3 and the autophagy factor Beclin-1 showed no significant changes. The results showed that the damage to neural structures and cognitive functions caused by *A. cantonensis* early infection was not caused by cell death.

Many studies have pointed out that immune-related cytokines/chemokines can affect cognitive function. Kipnis et al. injected T cells into nude mice, and their spatial memory learning ability was significantly improved [36]. Derecki et al. conducted a water maze test with C57BL/6 mice that had been knocked out of the IL-4 gene and found that their spatial memory learning ability was decreased [37]. In addition to the peripheral immune factors that affect the brain, microglia and astrocytes are widely discussed in neurodegenerative diseases. It is generally believed that the abnormal effects of microglia and astrocytes will cause abnormal protein accumulation and increase in oxidative stress, which in turn leads to damage to nerve structure and affects cognitive function [38]. Angiostrongyliasis is known to activate microglia and increase oxidative stress in the brain. The results of this research indicate that the excretory/secretory products of larvae may cause synaptic loss to affect cognitive function. The relationship between *A. cantonensis* infection and neurodegenerative diseases is a subject worthy of study.

## Conclusions

In this study, it was found that *A. cantonensis* caused synaptic loss in the early stage of infection. The infection affects the cognitive functions of the host and did not cause the death of neurons. Damage to nerve structure was not caused by the mechanical destruction of larval migration or pathological changes. Therefore, it was speculated that the larvae may cause synaptic loss through the ESP, thereby affecting the learning and memory abilities of the host. It is anticipated that the influence of synaptic loss caused by the infection of *A. cantonensis* may provide another possible explanation for the loss of cognitive functions postinfection.

## Supplementary Information

**Additional file 1: Fig. S1.** Evaluation of mobility of C57BL/6 mice before and after Angiostrongylus cantonensis infection by open field tests. a The total distance of C57BL/6 mice moved in open field tests. b The velocity of C57BL/6 mice in open field tests.

**Additional file 2: Fig. S2.** The full blots of PSD95, synaptophysin and NeuN expression in hippocampus and prefrontal cortex of mice infected with* A. cantonensis* detected by Western blotting. a b The level of PSD95, synaptophysin and NeuN expression in hippocampus and prefrontal cortex of mice infected with* A. cantonensis*.

## Data Availability

The data obtained from this study are included in this published article.

## Abbreviations

AD: Alzheimer's disease
ANOVA: Analysis of variance
BDNF: Brain-Derived Neurotrophic Factor
CNS: Central nervous system
DI: Discrimination index
ESP: Excretory/secretory product
IgA + G + M: Immunoglobulin A + G + M
IgG: Immunoglobulin G
IgM: Immunoglobulin M
IHC: Immunohistochemistry
IL-1β: Interleukin-1β
MWM: Morris water maze
NeuN: Neuronal nuclear
NF-κB: Nuclear factor-κB
NMDAR: N-methyl-d-aspartate receptor
NOR: Novel object recognition
PSD95: Postsynaptic density protein 95
RIP3: Receptor-interacting protein 3
SHH: Sonic hedgehog
SYN: Synaptophysin
TBI: Traumatic brain injury
TNF-α: Tumor necrosis factor-α
TrkB: Tropomyosin receptor kinase B
