# Supplementary figures and images for: Synaptic loss and progression in mice infected with Angiostrongylus cantonensis in the early stage
Source: J Neuroinflammation. 2022 Apr 12;19:85. doi: 10.1186/s12974-022-02436-8 (PMC9006624; doi:10.1186/s12974-022-02436-8)

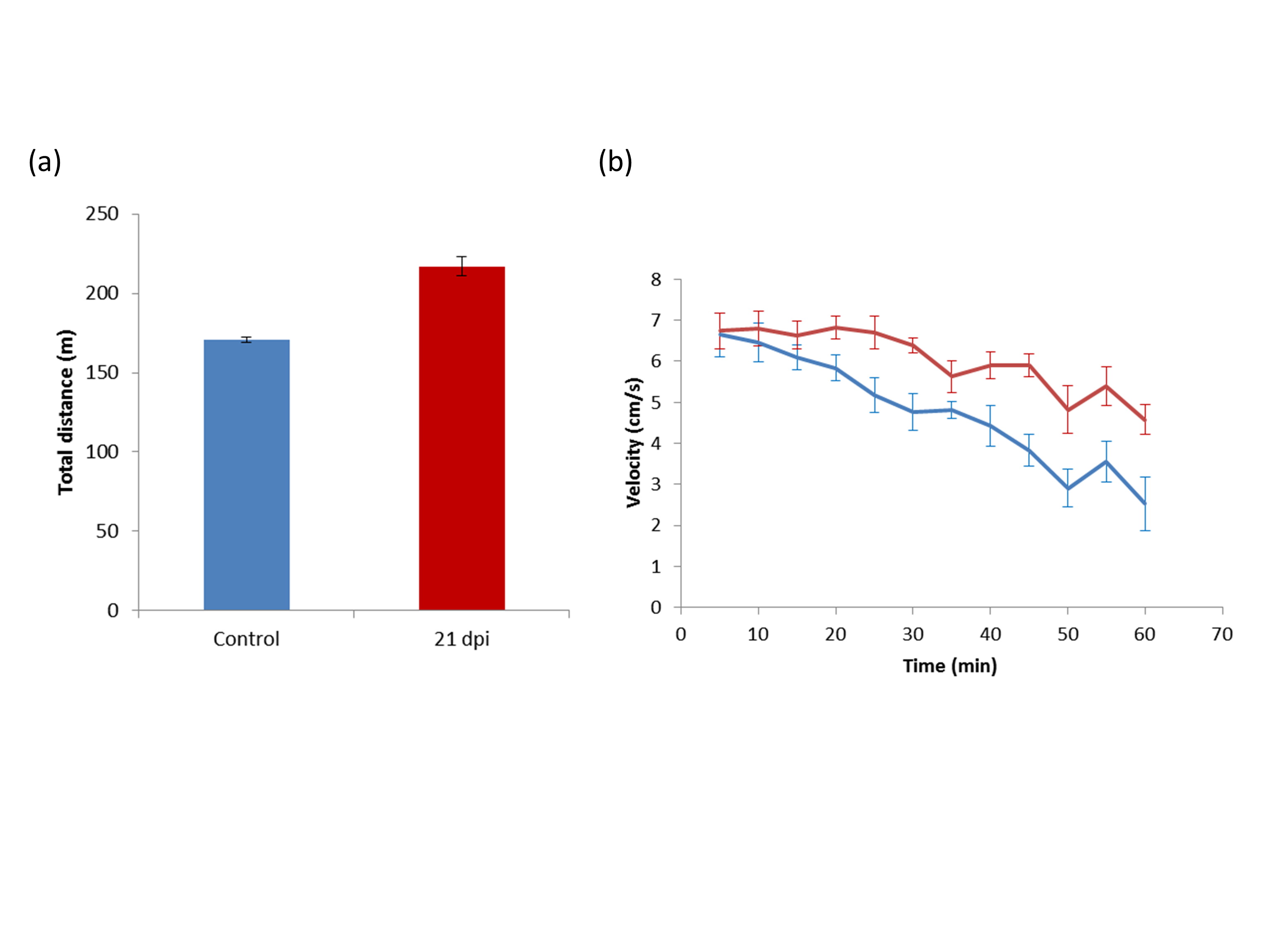

Supplement: Supplementary file 1 — Additional file 1: Fig. S1. Evaluation of mobility of C57BL/6 mice before and after Angiostrongylus cantonensis infection by open field tests. a The total distance of C57BL/6 mice moved in open field tests. b The velocity of C57BL/6 mice in open field tests. [file 12974_2022_2436_MOESM1_ESM.jpg]

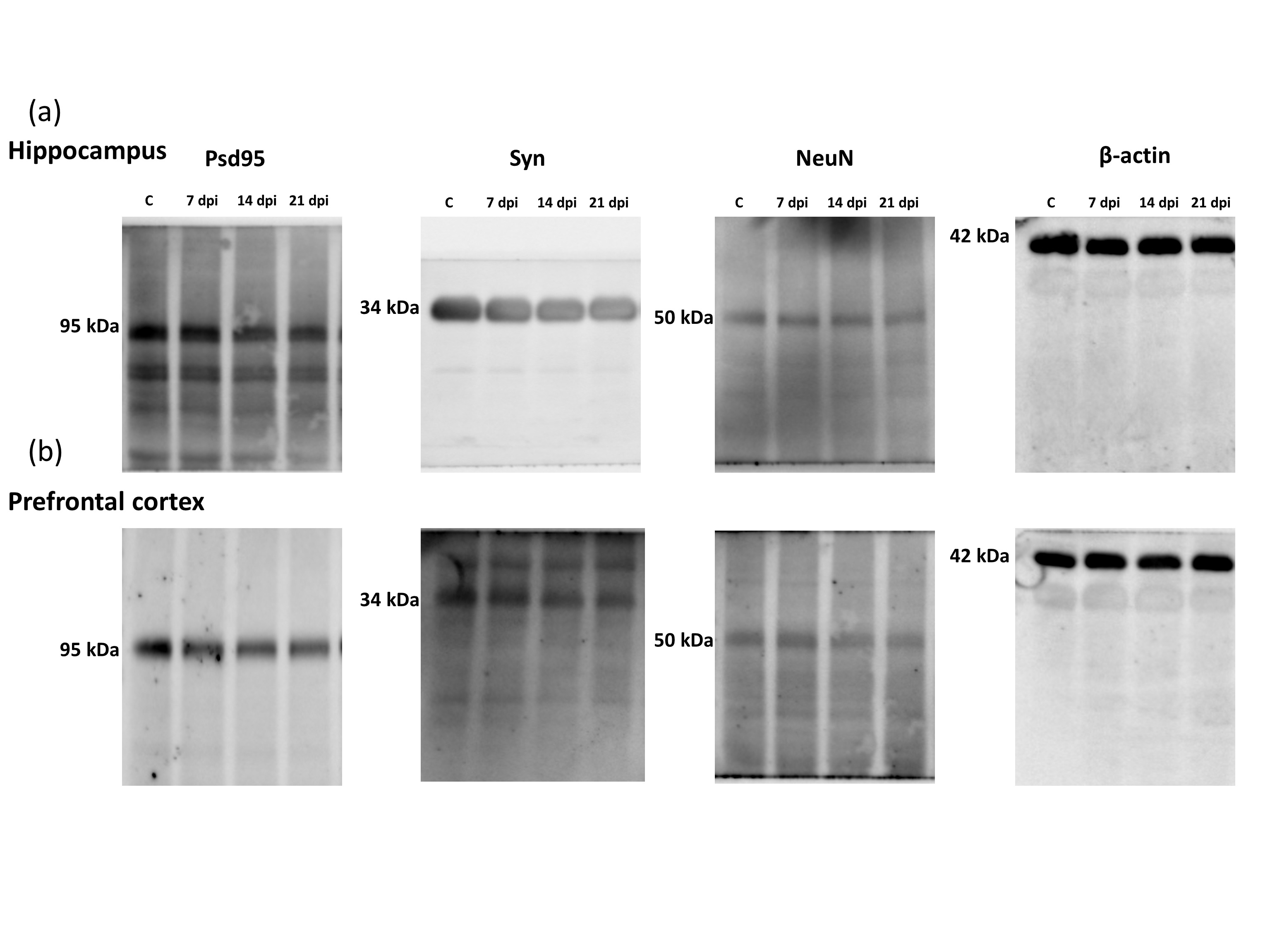

Supplement: Supplementary file 2 — Additional file 2: Fig. S2. The full blots of PSD95, synaptophysin and NeuN expression in hippocampus and prefrontal cortex of mice infected with A. cantonensis detected by Western blotting. a b The level of PSD95, synaptophysin and NeuN expression in hippocampus and prefrontal cortex of mice infected with A. cantonensis. [file 12974_2022_2436_MOESM2_ESM.jpg]
